# Mechanisms of Cisplatin-Induced Ototoxicity and Otoprotection

**DOI:** 10.3389/fncel.2017.00338

**Published:** 2017-10-27

**Authors:** Sandeep Sheth, Debashree Mukherjea, Leonard P. Rybak, Vickram Ramkumar

**Affiliations:** ^1^Department of Pharmacology, Southern Illinois University School of Medicine, Springfield, IL, United States; ^2^Department of Surgery (Otolaryngology), Southern Illinois University School of Medicine, Springfield, IL, United States

**Keywords:** cisplatin, ototoxicity, otoprotection, oxidative stress, apoptosis, antioxidants, anti-inflammatory agents

## Abstract

Evidence of significant hearing loss during the early days of use of cisplatin as a chemotherapeutic agent in cancer patients has stimulated research into the causes and treatment of this side effect. It has generally been accepted that hearing loss is produced by excessive generation of reactive oxygen species (ROS) in cell of the cochlea, which led to the development of various antioxidants as otoprotective agents. Later studies show that ROS could stimulate cochlear inflammation, suggesting the use of anti-inflammatory agents for treatment of hearing loss. In this respect, G-protein coupled receptors, such as adenosine A_1_ receptor and cannabinoid 2 receptors, have shown efficacy in the treatment of hearing loss in experimental animals by increasing ROS scavenging, suppressing ROS generation, or by decreasing inflammation. Inflammation could be triggered by activation of transient receptor potential vanilloid 1 (TRPV1) channels in the cochlea and possibly other TRP channels. Targeting TRPV1 for knockdown has also been shown to be a useful strategy for ensuring otoprotection. Cisplatin entry into cochlear hair cells is mediated by various transporters, inhibitors of which have been shown to be effective for treating hearing loss. Finally, cisplatin-induced DNA damage and activation of the apoptotic process could be targeted for cisplatin-induced hearing loss. This review focuses on recent development in our understanding of the mechanisms underlying cisplatin-induced hearing loss and provides examples of how drug therapies have been formulated based on these mechanisms.

## Cisplatin Ototoxicity

Cisplatin is a widely used and effective drug for the treatment of solid tumors ranging from ovarian, lung, head, and neck to testicular cancer. Dose limiting side effects, such as ototoxicity, neurotoxicity, and nephrotoxicity, are generally encountered with a majority of patients treated with cisplatin-based chemotherapy. Methods to increase diuresis, such as hydration, have been shown to reduce nephrotoxicity. However, to date no effective FDA approved treatment for ototoxicity is available. Cisplatin-induced hearing loss is primarily in the high frequency range. It is bilateral and permanent and severely affects the quality of life for cancer patients. The incidence of cisplatin-induced hearing loss in children ranges from 22 to 77% (Knight et al., 2005; Kushner et al., 2006; Coradini et al., 2007). This range reflects hearing loss observed with the different doses and duration of cisplatin treatment in addition to the different age groups of the patients who were treated. Accordingly, children show greater risk for developing hearing loss following cisplatin treatment than adults (Knight et al., 2005; Kushner et al., 2006; Gurney et al., 2007). Hearing loss is especially difficult for children who are undergoing treatment for brain tumors, such as neuroblastoma. It could affect early speech development and hamper social integration. Development of effective therapies for treating hearing loss is therefore of primary importance.

Several approaches have been undertaken over the past two decades to treat cisplatin ototoxicity. These include the use of localized or systemic administration of antioxidants or drugs which activate endogenous antioxidant systems. Cisplatin induces apoptosis of hair cells through activation of mitochondrial pathway which can be targeted to inhibit cell death. Another approach involves using anti-inflammatory agents which target the pro-inflammatory mechanisms associated with cisplatin treatment in the cochlea. Recent insights into the entry processes of cisplatin in hair cells and other cells in the cochlea should stimulate the development of drugs which specifically block entry of drugs into these cells without affecting cisplatin entry into cancer cells. This report reviews the currently accepted mechanisms underlying cisplatin-induced damage or death to cochlear cells and highlights how these mechanisms could guide the future development of effective otoprotective agents.

## Cochlear ROS Generation and Antioxidant Defense System

The normal function of the cochlea requires its high metabolic activity in areas such as the stria vascularis, spiral ligament, and spiral prominence (Salt et al., 1987; Ryan, 1988). This high metabolic activity leads to leakage of electrons from the mitochondrial respiratory chain which can react with oxygen (O_2_) to form superoxide (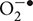). Environmental stimuli which increase the metabolic activity of the cochlea, such as loud noise, are expected to increase oxidative stress in the cochlea. The metabolic demand on the cochlea renders it very sensitive to hypoxic events and ischemia-reperfusion injuries (Seidman et al., 1991). Ototoxic drugs, such as cisplatin have been shown to increase the generation of reactive oxygen species (ROS) (Clerici and Yang, 1996; Kopke et al., 1997) by either stimulating enzyme systems linked to this process or by inactivating antioxidant systems (Church et al., 1995; Rybak et al., 1995). Rats injected with cisplatin demonstrate reduced cochlear glutathione (GSH) and antioxidant enzyme activities (Ravi et al., 1995). A primary target of cisplatin for generation of ROS is the NOX3 NADPH oxidase system (Banfi et al., 2004). NOX3 is induced by cisplatin and knockdown of this enzyme by *trans*-tympanic delivery of siRNA protects against cisplatin-induced ototoxicity (Mukherjea et al., 2010). Since NOX3 is localized primarily to the cochlea, systemic administration of inhibitors of NOX3 could effectively reduce enzyme activity and treat hearing loss. Another active ROS generating system in the cochlea is xanthine oxidase. This enzyme converts hypoxanthine (a metabolite derived from the breakdown of adenosine by adenosine deaminase) to uric acid. Inhibition of this enzyme by allopurinol contributes to reductions in cisplatin-induced ototoxicity and nephrotoxicity when administered with ebselen, a glutathione peroxidase (GSH.Px) mimetic (Lynch et al., 2005).

**FIGURE 1:**
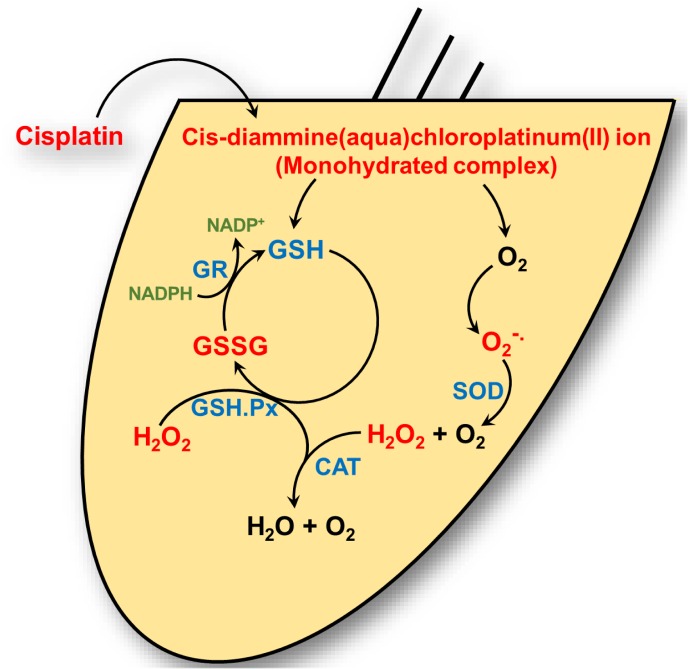
Cisplatin’s interaction with the cochlear antioxidant defense system. Cisplatin is converted to a *cis*-diammine(aqua)chloroplatinum(II) (a monohydrate cisplatin complex) upon entering the cell cytoplasm. These reactive platinum species can react with molecular oxygen (O_2_) to generate superoxide (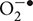) which is detoxified by superoxide dismutase (SOD) to hydrogen peroxide (H_2_O_2_) and oxygen. Hydrogen peroxide is further detoxified by catalase to water (H_2_O) and oxygen. Cisplatin reactive intermediates readily bind to and oxidizes the antioxidant reduced glutathione (GSH) to oxidized glutathione (GSSG). Glutathione peroxidase (GSH.Px) consumes GSH to produce GSSG in the process of converting H_2_O_2_ into H_2_O. Glutathione reductase (GR) reduces GSSG to GSH by using the reduced from of nicotinamide adenine dinucleotide phosphate (NADP^+^), NAPDH, as cofactor.

The cochlea possesses an efficient antioxidant defense system. This includes antioxidants such as vitamin C, vitamin E, and low molecular weight thiols, such as GSH (Kopke et al., 1999). Studies in the guinea pig cochlea show that the highest levels of GSH are present in the basal and intermediate cells of the stria vascularis and in cells of the spiral ligament (Usami et al., 1996). This distribution matches well with the distribution of glutathione *S*-transferase (el Barbary et al., 1993), an enzyme which conjugates and detoxifies xenobiotics (such as cisplatin). Increased levels of glutathione *S*-transferase in cancer cells can aid in the inactivation of cisplatin and contribute to resistance to cisplatin in the cochlea. In addition, the cochlea expresses several antioxidant enzymes, which include superoxide dismutase (SOD), GSH.Px, and catalase (CAT). SOD catalyzes the conversion of 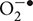 to H_2_O_2_ and O_2_ while CAT converts H_2_O_2_ to O_2_ and H_2_O. GSH.Px reduces H_2_O_2_ and possibly other peroxides. The enzyme GSH.Px also catalyzes the conversion of reduced GSH to its oxidized form (GSSG), in the process of detoxifying H_2_O_2_. In addition, glutathione reductase (GR) is important for the defense against ROS by aiding in the regeneration of GSH from GSSG (**Figure 1**). Two forms of SOD are expressed in the cochlea. A Cu/Zn isoform of SOD is localized in the cytosol, while a Mn-regulated isoform (Mn-SOD) is localized to the mitochondria (Yao and Rarey, 1996). Mn-SOD is localized to metabolically active sites in the cochlea such as stria vascularis, spiral ligament, spiral prominence, spiral limbus, and organ of Corti (Lai et al., 1996). Activities of SOD were higher in the subcellular fractions of stria vascularis and spiral ligament compared to the organ of Corti (Pierson and Gray, 1982; Yao and Rarey, 1996). High activities of other antioxidant enzymes were also observed in the lateral wall tissues compared to the rest of the cochlea, suggesting their roles in mitigating oxidative stress in these regions (Pierson and Gray, 1982). In the absence of this ROS detoxification system, ROS can produce cellular damage by lipid peroxidation, with increased levels of the lipid peroxide, malondialdehyde, and 4-hydroxynonenal.

## Increased ROS Generation Contributes to Cisplatin-Induced Hearing Loss

Exposure of explants to ROS induces bleb formation and changes in length of outer hair cells which were attenuated by co-administration of the antioxidant, deferoxamine, or antioxidant enzymes (Clerici et al., 1995). Infusion of ROS into the guinea pig ear led to increased compound action potential (CAP) threshold amplitudes, which were reduced by co-administration of antioxidant or antioxidant enzymes (Clerici and Yang, 1996). Furthermore, it was observed that cochleae obtained from cisplatin-treated animals showed depletion of GSH and reduced activities of antioxidant enzymes, such as SOD, CAT, GSH.Px, and GR along with increased evidence of lipid peroxidation (Rybak et al., 2000). This reduction in antioxidant capacity in the cochlea could result from: (1) covalent binding of cisplatin to sulfhydryl groups within the antioxidant enzymes, causing enzyme inactivation; (2) loss of metal cofactors, such as copper and selenium, which are vital for activity of SOD and GSH.Px; (3) increased ROS which could exhaust antioxidant enzymes; and/or (4) depletion of cochlear antioxidant enzyme cofactors, such as GSH and NADPH, which are essential for GSH.Px and GR activities, respectively (for review see Rybak et al., 2000). The increased oxidative stress within the cochlea could increase lipid peroxidation of membranes, inactivate essential cellular enzymes and membrane transporters, and disrupt ion channel function. The ultimate effect of increased ROS generation is to promote apoptotic and necrotic cell death. These data suggest that ROS plays a critical role in cisplatin-induced ototoxicity and that its inhibition could ameliorate hearing loss.

As discussed below, one important result of increased oxidative stress is induction of inflammatory processes in the cochlea. The importance of managing ROS in mediating otoprotection is underscored by the observation that polymorphisms of glutathione *S*-transferase gene increase susceptibility to cisplatin hearing loss (Oldenburg et al., 2007). In this report, it was shown that patients inheriting the 105Ile/105Ile-GSTP1 or 105Val/105Ile-GSTP1 alleles had greater hearing loss than those inheriting the 105Val/105Val-GSTP1 alleles (Oldenburg et al., 2007).

## Targeting Oxidative Stress for Treating Cisplatin-Induced Hearing Loss

The cochlea is endowed with a number of distinct cellular mechanisms which could contribute to otoprotection. These include various endogenous antioxidant enzymes and antioxidants (detailed above), heat shock proteins, kidney injury molecule-1, anti-apoptotic proteins, transcription factors such as nuclear factor erythroid 2-related factor 2 (Nrf2) and signal transducer and activator of transcription (STAT) proteins, and a number of hormone and G-protein-coupled receptors (Ford et al., 1997; Oh et al., 2000; Mukherjea et al., 2006; So et al., 2006; Borse et al., 2017). Therefore, it is very surprising that these mechanisms are not able to effectively protect against cisplatin ototoxicity. However, it is possible that over the course of cisplatin treatment in animal models and humans that these protective systems become overwhelmed and are no longer able to manage the toxicity. It is believed that exogenously administered antioxidants or other drugs can boost the local protective mechanism to ensure otoprotection.

Early studies were directed at examining the effects of drugs which reduce oxidative stress for treating hearing loss. Studies performed in animals indicated that antioxidants protected against cisplatin-induced hearing loss. Many of these antioxidants are thiol compounds which have high affinities for platinum. This forms the basis for their protection against cisplatin toxicity but also could account for the antitumor interference with cisplatin. Studies showed that *N*-acetyl cysteine (NAC) protected against cisplatin-ototoxicity in rats (Dickey et al., 2008) and guinea pigs (Choe et al., 2004). Sodium thiosulfate (STS) was also effective against cisplatin-induced hearing loss (Otto et al., 1988). However, systemic administration of this drug led to formation of cisplatin–STS complex, which reduced the levels of cisplatin in circulation needed for effective antitumor therapy (Wimmer et al., 2004). Thus, local application of STS into the cochlea would be required to provide effective otoprotection (Wimmer et al., 2004), as was shown previously (Wang et al., 2003). Several studies have shown that D-methionine, another sulfur-containing compound, protects against cisplatin-induced hearing loss when administered by both the systemic and local routes (Campbell et al., 1996; Korver et al., 2002). At the cellular level, the otoprotection mediated by this compound was associated with its ability to increase the activity of intrinsic antioxidant enzymes (Campbell et al., 2003). Other antioxidants which show promise against cisplatin-induced hearing loss include ebselen, lipoic acid, diethyldithiocarbamate, and 4-methylthiobenzoic acid (Rybak et al., 1999). Similarly, high doses of amifostine provided otoprotection in hamsters but its use was associated with neurotoxicity (Church et al., 2004).

Contrary to the pre-clinical findings, studies performed in humans showed that STS protected against cisplatin-induced nephrotoxicity (Goel et al., 1989) but was ineffective against cisplatin-induced hearing loss (Zuur et al., 2007). Amifostine was also ineffective as an otoprotectant in patients with metastatic melanoma (Ekborn et al., 2002) and in children suffering from neuroblastoma or germ cell tumors and were on a chemotherapeutic regimen including cisplatin (Marina et al., 2005; Sastry and Kellie, 2005). However, later studies showed that higher doses of amifostine were able to provide significant otoprotection (Fouladi et al., 2008).

**FIGURE 2:**
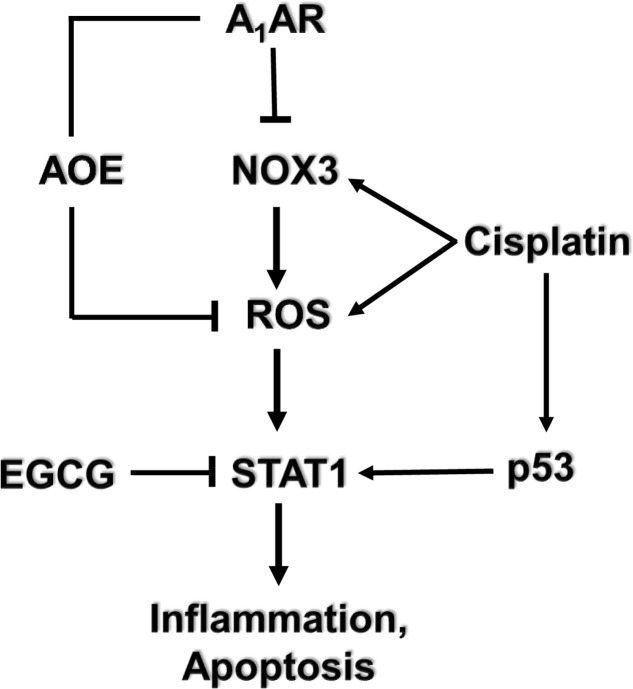
Mechanism of cisplatin-induced hearing loss and A_1_ adenosine receptor (A_1_AR)-dependent otoprotection. Cisplatin mediates NOX3 activation and reactive oxygen species (ROS) generation. The generation of ROS promotes signal transducer and activator of transcription 1 (STAT1) activation which stimulates the inflammatory process. Activated STAT1 association with active p53 promotes the apoptosis of cochlear cells. The otoprotective effects of A_1_AR activation is mediated by reducing oxidative stress in the cochlea by activating antioxidant enzymes (AOE) and/or by suppressing the induction of NOX3. EGCG, a known inhibitor of STAT1, has been shown to protect against cisplatin-induced hearing loss.

Several G-protein-coupled receptors have been characterized in the cochlea, activation of which confer protection against cisplatin-induced hearing loss. Immunolabeling studies show distribution of the A_1_ adenosine receptors (A_1_ARs) in the stria vascularis, spiral ganglion cells, and organ of Corti (Vlajkovic et al., 2009). In the organ of Corti, the greatest distribution of A_1_AR is in the inner hair cells, Deiter’s cells and lower levels in the outer hair cells. Expression of A_1_AR was also observed in mouse organ of Corti-derived cell lines including the UB/OC-1 cells (Kaur et al., 2016) and HEI-OC1 cells (unpublished data). Localized application of A_1_AR agonist resulted in an increase in the activities of the antioxidative enzymes GSH.Px and SOD (Ford et al., 1997). Furthermore, A_1_AR agonist also reduced the cisplatin-mediated increase in malondialdehyde levels in the cochlea resulting in protection against cisplatin-induced hair cell damage and hearing loss (Whitworth et al., 2004). No significant benefits was observed following activation of the A_2a_AR and A_3_AR (Whitworth et al., 2004) which are also distributed in the cochlea (Vlajkovic et al., 2009). Systemic administration of the adenosine amine congener (ADAC) was shown to protect against cisplatin ototoxicity, presumably by activating the A_1_AR (Gunewardene et al., 2013). Recent data support an anti-inflammatory role of A_1_AR activation in the cochlea mediated by suppression of the NOX3 isoform of NADPH oxidase and suppression of STAT1-mediated inflammatory pathway (Kaur et al., 2016). STAT1 activation plays an integral role in cisplatin ototoxicity, as inhibition or knockdown of this transcription factor reduced cisplatin-induced hearing loss (Schmitt et al., 2009; Kaur et al., 2011; Mukherjea et al., 2011). A recent study from our laboratory has further documented an essential role of STAT1 in mediating cisplatin-induced hearing loss, as inhibition of this factor by a green tea extract and a known STAT1 inhibitor, epigallocatechin-3-gallate (EGCG), provided otoprotection (Borse et al., 2017). In a rodent model, EGCG protected against cisplatin-induced hearing loss without compromising cisplatin antitumor efficacy (Borse et al., 2017) (see model depicted in **Figure 2**). *In vitro* studies performed in HEI-OC1 cells demonstrate that cannabinoid 2 receptor (CB2) agonists reduce cisplatin-induced cell killing (Jeong et al., 2007). CB2 are also expressed in the stria vascularis, inner hair cells and spiral ganglion cells of the cochlea from adult albino rats (Martin-Saldana et al., 2016). Recent studies from our laboratories support an otoprotective role of CB2 activation in the cochlea, which is mediated at least in part, through inhibition of STAT1 (Ghosh et al., 2016; unpublished data). Thus, the protective action of CB2 could share a similar mechanism as observed by A_1_AR, namely inhibition of STAT1.

**FIGURE 3:**
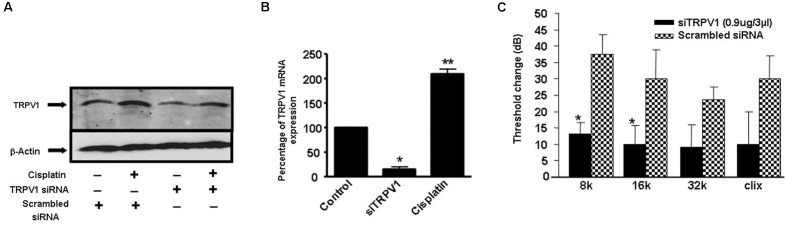
Round window administration of siRNA against *TRPV1* protects against cisplatin-induced ototoxicity in rats. **(A)** Round window application of *TRPV1* siRNA reduced both, basal and cisplatin-stimulated TRPV1 protein levels in the cochlea, assessed 24 h following cisplatin administration. **(B)** *TRPV1* siRNA suppressed *TRPV1* expression in the rat cochlea. **(C)** Functional studies show that *TRPV1* siRNA (0.9 μg) administered by round window application protected against cisplatin-induced elevations in hearing thresholds at all frequencies tested and for click stimuli. Cisplatin (13 mg/kg i.p.) was administered 48 h following siRNA or a scrambled siRNA sequence and post-treatment ABRs were determined after an additional 72 h period. ^∗^*p* < 0.05 versus scrambled siRNA-treated cochleae and ^∗∗^*p* < 0.05 versus TRPV1 siRNA (*n* = 5). This figure was adapted from Mukherjea et al. (2008), with permission.

Additional studies from our laboratory implicated transient receptor potential vanilloid 1 (TRPV1) channels in cisplatin-mediated ototoxicity (Mukherjea et al., 2008). In a rat model, we showed knockdown of these channels by *trans*-tympanic administration of short interfering (si) RNA protected against cisplatin-induced hearing loss and damage to the outer hair cells (Mukherjea et al., 2008) (**Figure 3**). Protection was likely mediated by reducing the expression of a downstream target of TRPV1, such as NOX3, activation of which promotes ROS generation and STAT1 activation, as indicated above. STAT1 can promote both the inflammatory and pro-apoptotic actions of cisplatin in the cochlea (Kaur et al., 2011). This study suggests that inhibition of TRPV1 or NOX3 could serve as useful approaches for reducing cisplatin ototoxicity.

Characterization of cisplatin-induced cell death in HEI-OC1 cells showed induction of apoptosis by increased lipid peroxidation and altered mitochondrial permeability transition. It was shown that the calcium-channel blocker, flunarizine, attenuated cisplatin-induced cell death (So et al., 2006). The mechanism underlying the otoprotective action of flunarizine appears to involve activation of Nrf2 and increased expression of hemeoxygenase-1 (HO-1) (So et al., 2006). Flunarizine also exhibited an anti-inflammatory role, as evidenced from its ability to inhibit the ERK1/2 MAP kinase-nuclear factor (NF)-κB-dependent pathway (So et al., 2008).

## Mitochondrial Targets of Cisplatin-Induced Ototoxicity

### Bcl-2 Family

The Bcl-2 family of proteins consists of members that form the mitochondrial apoptotic pathway and function as regulators of cell death and cell survival. Among its members, Bcl-2 and Bcl-xL promote cell survival, whereas Bax, Bak, Bcl-XS, Bid, Bad, and Bim induce apoptosis (Siddiqui et al., 2015). The balance between the pro-apoptotic and anti-apoptotic proteins is crucial for the well-being of the cell. However, cellular damage caused by noxious stimuli can tilt this balance in favor of apoptosis. This process is initiated when pro-apoptotic protein such as Bax and Bid translocate from the cytoplasm to the mitochondria. This triggers a sequence of events leading to the permeabilization of the outer mitochondrial membrane, which results in the loss of mitochondrial membrane potential, generation of ROS, and release of cytochrome c from mitochondria into the cytoplasm (Siddiqui et al., 2015).

**FIGURE 4:**
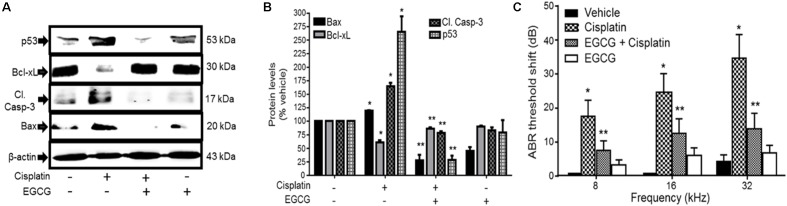
Epigallocatechin-3-gallate (EGCG) inhibited cisplatin-induced apoptosis and hearing loss. **(A)** UB/OC-1 cells pretreated with either vehicle or EGCG (100 μM) followed by cisplatin (20 μM) for 24 h were analyzed for pro-apoptotic proteins, such as p53, cleaved caspase-3 and Bax and anti-apoptotic protein, Bcl-xL. The expression of pro-apoptotic proteins was substantially reduced by EGCG while the reductions in Bcl-xL were attenuated. **(B)** The bar graph represents results from **A** after normalization with β-actin bands and are presented as the mean ± SEM. ^∗^*p* < 0.05 versus vehicle and ^∗∗^*p* < 0.05 versus vehicle + cisplatin (*n* = 4). **(C)** Pre-treatment ABR thresholds were recorded in Wistar rats, which were then treated with oral EGCG (100 mg/kg body weight). Cisplatin (11 mg/kg) was administered intraperitoneally 24 h later and animals were continued on daily oral EGCG treatments for additional 3 days. Post-treatment ABRs were performed on day 4. Daily oral administration of EGCG protected from cisplatin-induced ABR threshold shifts at all frequencies tested. ^∗^*p* < 0.05 versus vehicle and ^∗∗^*p* < 0.05 versus vehicle + cisplatin (*n* = 4). This figure was adapted from Borse et al. (2017), with permission.

Several studies have implicated the mitochondrial pathways in the apoptosis of auditory cells after cisplatin treatment. Mongolian gerbils administered cisplatin showed deterioration in the responses of both distortion product otoacoustic emissions (DPOAE) and the endocochlear potential as compared with age-matched controls (Alam et al., 2000). The cisplatin-induced hearing loss was correlated with increased levels of Bax and decreased expression of Bcl-2 in the cells of organ of Corti, spiral ganglion and the lateral wall, as determined by immunohistochemistry. Moreover, cisplatin significantly elevated the ratio of Bax to Bcl-2, an important indicator of apoptosis, in the three representative regions in all turns of the cochlea. Similarly, cochlear hair cells from cisplatin-treated guinea pigs demonstrated apoptosis which was linked to the activation and redistribution of cytosolic Bax and the release of cytochrome c from the mitochondria (Wang et al., 2004). In another study conducted in HEI/OC1 cells, cisplatin-induced apoptosis through mitochondrial pathway which involved truncation of Bid, mitochondrial translocation of Bax, and release of cytochrome c (Devarajan et al., 2002). Recently, cisplatin treatment in UB/OC-1 cells significantly increased expression of *Bax*, but reduced the levels of *Bcl-xL* (Borse et al., 2017). Treatment with EGCG, a known inhibitor of STAT1, reversed these cisplatin-mediated effects on the expression of *Bax* and *Bcl-xL* (Borse et al., 2017) and protected against hearing loss (**Figure 4**). These data indicate that cisplatin induces apoptosis through activation of mitochondrial pathway and that inhibiting elements of this pathway could alleviate hearing loss.

### Caspases

Stress signals from the mitochondria also regulate the cleavage of pro-caspase-9 into its active form, caspase-9 (Hengartner, 2000). Activated caspase-9 can then cleave and activate the downstream effector caspase, caspase-3, resulting in apoptotic destruction of the cell. Caspase-3 is also activated by caspase-8, which is an initiator caspase activated by plasma membrane death receptors (Salvesen and Dixit, 1997). Cisplatin induces apoptosis of auditory hair cells and cochlear cell lines via activation of initiator caspase-9 and its effector caspase-3. Cisplatin-induced activation of caspase-9 and caspase-3 was also seen in HEI/OC1 (Devarajan et al., 2002; Chung et al., 2008) cells and UB/OC1 cells (Borse et al., 2017). Wang et al. (2004) reported the activation of caspase-9 and caspase-3, but not caspase-8, in cochlear hair cells of pigmented guinea pigs treated with cisplatin. Furthermore, intracochlear perfusion of specific inhibitors of caspase-9 and caspase-3 protected against cisplatin-induced hair cell death and hearing loss in these animals (Wang et al., 2004). In another study, treatment of neonatal rat organ of Corti explants with caspase-1- and caspase-3-specific inhibitors and a general caspase inhibitor protected more than 80% of the auditory hair cells from cisplatin-induced apoptosis (Liu et al., 1998). Unlike other caspases, caspase-1 is not linked with the induction of apoptosis in cells. Caspase-1 is studied for its role in initiating inflammatory immune responses through formation of inflammasomes. Activation of caspase-1 was recently reported to induce hearing loss after cytomegalovirus (CMV) infection in the inner ear through upregulation of its downstream inflammatory factors, such as interleukin-1β and interleukin-18 (Shi et al., 2015). Therefore, it is possible that caspase-1-specific inhibitor may protect from cisplatin ototoxicity by inhibiting cisplatin-induced inflammation in the cochlea.

Several mechanisms underlying cisplatin-induced caspase activation has been proposed. For example, cisplatin-induced apoptosis and caspase-3 activation in HEI/OC1 cells was reduced after treatment with NF-κB inhibitors, Bay 11-7085 and SN-50 (Chung et al., 2008). This finding indicates that NF-κB is activated by cisplatin and plays a pro-apoptotic role in HEI/OC1 cell death. Cisplatin-induced apoptosis and activation of caspase-3, -8, and -9 were also inhibited by activation of CB2 receptors (Jeong et al., 2007), which are present in the cochlear cells and upregulated by cisplatin (Martin-Saldana et al., 2016). Cisplatin-mediated expression of cleaved-caspase-3 in UB/CO1 cells was inhibited by treatment with EGCG through inhibition of STAT1 (Borse et al., 2017). Taken together, loss of auditory cells as a result of cisplatin-induced apoptosis involves activation of caspases as a final common pathway activated by different upstream signaling pathways described above. Inhibition of one or more of these signaling pathways could effectively rescue hair cell loss and restore hearing.

### p53

The general mechanism of action of cisplatin in tumor cells is that it forms crosslinks with the purine bases of the DNA, causing DNA damage. The overwhelming DNA damage caused by formation of DNA adducts interferes with the DNA replication and repair mechanisms, which subsequently induces apoptosis (Dasari and Tchounwou, 2014). The apoptotic signal is initiated by activation (through phosphorylation) of p53, a tumor suppressor gene and an important mediator of DNA damage-induced apoptosis. Accumulating evidence suggest that in response to stress signal, a fraction of activated p53 rapidly translocates to the mitochondria, where it interacts with the various pro- and anti-apoptotic members of Bcl-2 family to either activate or inhibit them, respectively (Vaseva and Moll, 2009). In the mitochondria, p53 also induces loss of mitochondrial membrane potential, cytochrome c release and caspase-3 activation, triggering apoptosis (Marchenko et al., 2000). These findings were consistent with those in p53-deficient cells, where Bax translocation, cytochrome c release, and caspase-3 activation were downregulated, confirming that p53 acts upstream of mitochondrial apoptotic regulators (Morris et al., 2001). Interestingly, overexpression of anti-apoptotic Bcl-2 or Bcl-xL prevented stress signal-mediated mitochondrial accumulation of p53 and apoptosis, suggesting a feedback loop between p53 and mitochondrial apoptotic regulators (Marchenko et al., 2000).

The role of p53 in the regulation of cisplatin-induced apoptosis in the auditory system has been investigated. Application of the p53 inhibitor, pifithrin-α, to cochlear organotypic cultures exposed to cisplatin attenuated hair cell damage (Zhang et al., 2003; Benkafadar et al., 2017). The protection was associated with reduction in the expression of *p53*, *caspase-1*, and *caspase-3* (Zhang et al., 2003). The validity of these *in vitro* results was tested in hair cells derived from p53-deficient mice, which exhibited resistance to cisplatin-induced apoptosis (Benkafadar et al., 2017) and reduced caspase-3 activation (Cheng et al., 2005a,b). Although systemic inhibition of p53 protects against cisplatin-induced ototoxicity, this treatment strategy would interfere with the anti-cancer efficacy of cisplatin, restricting its use for treatment in humans. To overcome this problem, Benkafadar et al. (2017) demonstrated that intra-tympanic application of pifithrin-α protects auditory function without compromising the chemotherapeutic efficacy of systemically administered cisplatin. They further showed that systemic administration of pifithrin-α even sensitizes TP53-mutant tumors to cisplatin. These results illustrate the role of p53 as an important regulator of cisplatin-induced apoptosis in auditory hair cells.

## Cisplatin Targets DNA in the Cochlea

Previous studies have shown that platinated DNA accumulates in the nuclei of outer hair cells, supporting cells, marginal cells of the stria vascularis and cells in the spiral ligament (van Ruijven et al., 2005). Other studies have also shown DNA adduct in spiral ganglion neurons following cisplatin administration. The cochlear cells express DNA repair enzymes which can reduce the level of DNA adduct formed over time. These repair enzymes, known as nucleotide excision repair (NER) enzymes, are classified as transcriptional-coupled repair (TCR) enzymes or global DNA repair (GDR) enzymes which differ in their targets. Defective TCR function is observed in Cockayne syndrome which is characterized by progressive hearing loss. Outer hair cells from Cockayne syndrome group A (*Csa*^-^/^-^) and group B (*Csb*^-^/^-^) mice were hypersensitive to cisplatin. In contrast, *Xpc*^-^/^-^ mice which were deficient in global genome repair enzymes showed normal sensitivity to cisplatin (Rainey et al., 2016). This study implicates DNA damage as one mechanism of cisplatin-induced loss of outer hair cells and hearing loss and suggests that TCR plays a primary role in protecting hair cells from cisplatin-induced hearing loss. The effects of eight single-nucleotide polymorphisms in excision repair cross-complementing group 1, 2, 4, and 5 (*ERCC1*, *ERCC2*, *ERCC4*, and *ERCC5*) genes and xeroderma pigmentosum complementary group C and A (*XPC* and *XPA*) genes were assessed in patients with osteosarcoma who were treated with cisplatin. These studies showed that polymorphisms in DNA repair gene, *XPC*, was associated with increased cisplatin-induced ototoxicity in cancer patients. Increased ototoxicity was associated with the CC genotype of *XPC* Lys939Gln (Caronia et al., 2009).

## Uptake of Cisplatin Into the Cochlea

One of the major entry ports for cisplatin in the cochlea is the mammalian copper transport 1 (Ctr1). Ctr1 is highly expressed in the cochlea where it is localized to outer hair cells, inner hair cells, stria vascularis, and spiral ganglion neurons and contributes to drug entry and cell apoptosis. Decreasing cisplatin entry by intra-tympanic administration of copper sulfate, a substrate of Ctr1, protects against hearing loss induced by cisplatin (More et al., 2010). Cisplatin entry into cochlear cells is also mediated by organic cation transporter (OCT). Three isoforms of this protein exists, OCT1-3, which are present mainly in the kidneys and liver. Expression of OCT2 has also been detected in the organ of Corti and stria vascularis (Ciarimboli et al., 2010). Inhibition of these transporters with cimetidine protects against cisplatin-induced nephrotoxicity and ototoxicity. *OCT* knockout mice exhibit reduced toxicity to cisplatin. Single-nucleotide polymorphism in *OCT-2* gene protects against ototoxicity in children (Lanvers-Kaminsky et al., 2015). Several studies have reported that the entry of aminoglycosides into cochlear hair cells is mediated by mechanotransducer (MET) channels (Gale et al., 2001; Marcotti et al., 2005; Dai et al., 2006; Wang and Steyger, 2009; Alharazneh et al., 2011). In fact, changes in the structure of aminoglycosides which limits their entry through MET channels are less ototoxic (Huth et al., 2015). Cisplatin has also shown to block MET channels in chick cochlear hair cells (Kimitsuki et al., 1993). Recent studies have indicated that cisplatin-induced damage to hair cells in the zebrafish is dependent on functional MET channels (Thomas et al., 2013). These investigators showed that inhibition of mechanotransduction channels by quinine or EGTA protected against cisplatin-induced hair cell death. Furthermore, these investigators showed that zebrafish mutants which lacked mechanotransduction channels were also resistant to cisplatin-induced hair cell death. These studies suggest that mechanotransduction channels are a major contributor to the entry of cisplatin into hair cells, at least in the zebrafish. Interestingly, chemical inhibition of Ctr1 and OCT-2 did not provide significant protection against killing of lateral line hair cells in zebrafish (Thomas et al., 2013). Preliminary data from our lab indicate a similar pathway of entry of transplatin, the inactive isomer of cisplatin, which protects against cisplatin ototoxicity (Dhukhwa et al., 2017). These studies suggest that MET channels could serve as an additional entry ports for cisplatin into cochlear hair cells. Several other ion channels might also contribute to aminoglycoside uptake into hair cells. In addition to TRPV1, several TRP channels including TRPV4, TRPA1, TRPC3, and TRPML3 are expressed in the cochlea (Cuajungco et al., 2007; Asai et al., 2010). These channels have been shown to allow entry of aminoglycoside into kidney cells (Myrdal and Steyger, 2005). The potential role of these channels in mediating the entry of aminoglycosides and cisplatin into hair cells has not yet been established.

Megalin is a low-density lipoprotein which is highly expressed in the kidney and in the stria vascularis of the cochlea which accumulate high levels of platinum DNA adducts. Patients who showed cisplatin-induced hearing impairment demonstrate a higher frequency of megalin gene polymorphism compared to those with no hearing loss after cisplatin therapy. These findings implicate *megalin* gene polymorphisms in susceptibility to cisplatin ototoxicity (Riedemann et al., 2008). In the kidneys, megalin has been shown to bind β_2_-microglobulin, cytochrome c, retinal binding proteins, and polybasic antibiotics (such as gentamicin). These investigators show that the accumulation of cisplatin in the renal proximal tubules is mediated by binding of cisplatin-metallothionein complex to megalin (Klassen et al., 2004). The expression of *megalin* in the stria vascularis might similarly allow the accumulation of cisplatin into the stria vascularis.

## Conclusion

Studies described above have identified a number of different mechanisms which mediated cisplatin ototoxicity and provide the basis for rational drug design to treat this debilitating condition. While most of the drugs which target these mechanisms have shown promise, efficacy studies are still in the experimental animal stage (see **Table 1** for list of drugs/drug targets). These studies need to be extended in human clinical trials for final validation. The routes of drug administration would be a major issue, as systemic administration of these drugs could compromise cisplatin chemotherapeutic efficacy. In this regard, the use of a number of cisplatin transporters (namely Ctr1 and OCT2) and antioxidants would have the greatest likelihood of interfering with cisplatin antitumor efficacy (as discussed above). Localized delivery of these drugs into the cochlea would eliminate potential systemic toxicities but would require an additional minor surgical procedure to accomplish this goal. This would allow the use of a majority of the agents listed in this table for protection against hearing loss. Several of these drugs are currently in clinical use or in clinical trials for other indications. These include TNF-α antagonists which are used for the treatment of chronic inflammatory diseases (Aggarwal et al., 2012) and EGCG which is in various clinical trials for the treatment of cancer (Singh et al., 2011). These agents which are effective systemically could serve as initial candidates for treating cisplatin-induced hearing loss and other forms of hearing loss. Other drugs which could be advanced quickly into clinical use are MET channel blockers, such as bulky aminoglycoside antibiotics, which demonstrate low potential for ototoxicity (Huth et al., 2015). Continued research in this area would uncover new mechanisms underlying cisplatin-induced hearing loss and validate novel targets and drugs to treat this condition.

**Table 1:** Potential drug targets for treatment of cisplatin ototoxicity.

Drug targets	Mechanism(s)	Reference
**GPCRs**		
Adenosine A_1_ receptor (A_1_AR)	(1) Enhance endogenous antioxidant defense system	Ford et al., 1997; Whitworth et al., 2004
	(2) Suppression of NOX3/STAT1 inflammatory pathway	Kaur et al., 2016
Cannabinoid 2 receptor (CB2)	Anti-apoptotic	Jeong et al., 2007
**Pro-inflammatory markers**		
Transient receptor potential vanilloid 1 (TRPV-1)	(1) Marker for oxidative stress and inflammation in the cochlea	Mukherjea et al., 2008
	(2) Facilitates entry of cisplatin?	
Tumor necrosis factor-α (TNF-α)	Pro-inflammatory cytokine induced by cisplatin	So et al., 2007
Signal transducer and activator of transcription-1 (STAT1)	Pro-inflammatory transcription factor	Schmitt et al., 2009
Nuclear factor-κB (NF-κB)	Pro-inflammatory/pro-apoptotic transcription factor	Chung et al., 2008; So et al., 2008
**Transporters**		
Organic cation transporter 2 (OCT2)	Involved in cellular uptake mechanisms for cisplatin	Ciarimboli et al., 2010
Copper transport 1 (Ctr1)	Facilitates cisplatin entry into cells	More et al., 2010
Mechano-electrical transduction (MET) channel	Facilitates cisplatin entry into zebrafish lateral line	Thomas et al., 2013
**Antioxidant defense system**		
NOX3	Responsible for ROS generation in the cochlea	Banfi et al., 2004
Superoxide dismutase (SOD)	Detoxifies superoxide anion into H_2_O_2_ and O_2_	Ravi et al., 1995
Catalase (CAT)	Breaks down H_2_O_2_ into H_2_O and O_2_	Ravi et al., 1995
Glutathione (GSH)	Endogenous antioxidant molecule	Usami et al., 1996
Glutathione peroxidase (GSH.Px)	Catalyzes breakdown of H_2_O_2_ into H_2_O and O_2_ by using GSH	Ravi et al., 1995
Glutathione reductase (GR)	Converts oxidized glutathione (GSSG) to reduced GSH	Ravi et al., 1995
Glutathione *S*-transferase (GST)	Conjugates GSH with xenobiotics	el Barbary et al., 1993
Heme oxygenase-1 (HO-1)	Induced in response to oxidative stress	So et al., 2006
Nuclear factor erythroid 2-related factor 2 (Nrf2)	Regulator of cellular resistance to oxidants	So et al., 2006
Kidney injury molecule-1 (KIM-1)	Marker for oxidative stress in the cochlea	Mukherjea et al., 2006
Vitamin E	Antioxidant molecule	Kalkanis et al., 2004
*N*-acetyl cysteine (NAC)	Antioxidant molecule	Choe et al., 2004; Dickey et al., 2008
Sodium thiosulfate (STS)	Antioxidant molecule	Otto et al., 1988
D-Methionine (D-Met)	Antioxidant molecule	Campbell et al., 1996; Korver et al., 2002
Amifostine	Free radical scavenger	Church et al., 2004
Ebselen	Glutathione peroxidase mimetic	Rybak et al., 1999; Lynch et al., 2005
Allopurinol	Xanthine oxidase inhibitor	Lynch et al., 2005
**Miscellaneous**		
Heat shock protein 70 (HSP70)	Molecular chaperones important for protein folding	Roy et al., 2013; Baker et al., 2015
Signal transducer and activator of transcription-3 (STAT3)	Cytoprotection	Borse et al., 2017
Pifithrin-α	p53 inhibitor	Zhang et al., 2003; Benkafadar et al., 2017
Epigallocatechin-3-gallate (EGCG)	STAT1 inhibitor	Borse et al., 2017
Transcription-coupled repair (TCR)	Nucleotide excision repair (NCR) mechanism for damaged DNA	Rainey et al., 2016

## Author Contributions

SS and VR conceived and together outlined this review. SS and VR wrote the manuscript. DM and LR critiqued and revised the manuscript.

## Conflict of Interest Statement

The authors declare that the research was conducted in the absence of any commercial or financial relationships that could be construed as a potential conflict of interest.
